# AI for Doctors—A Course to Educate Medical Professionals in Artificial Intelligence for Medical Imaging

**DOI:** 10.3390/healthcare9101278

**Published:** 2021-09-28

**Authors:** Dennis M. Hedderich, Matthias Keicher, Benedikt Wiestler, Martin J. Gruber, Hendrik Burwinkel, Florian Hinterwimmer, Tobias Czempiel, Judith E. Spiro, Daniel Pinto dos Santos, Dominik Heim, Claus Zimmer, Daniel Rückert, Jan S. Kirschke, Nassir Navab

**Affiliations:** 1 Department of Neuroradiology, Klinikum rechts der Isar, School of Medicine, Technical University of Munich, D-81675 Munich, Germany; b.wiestler@tum.de (B.W.); martin.gruber@tum.de (M.J.G.); claus.zimmer@tum.de (C.Z.); jan.kirschke@tum.de (J.S.K.); 2 Computer Aided Medical Procedures, Technical University of Munich, D-81675 Munich, Germany; matthias.keicher@tum.de (M.K.); hendrik.burwinkel@tum.de (H.B.); Florian.Hinterwimmer@tum.de (F.H.); tobias.czempiel@tum.de (T.C.); dominik.heim@tum.de (D.H.); nassir.navab@tum.de (N.N.); 3 Institute for Artificial Intelligence and Informatics in Medicine, Technical University of Munich, D-81675 Munich, Germany; daniel.rueckert@tum.de; 4 Department of Radiology, University Hospital, LMU Munich, D-80336 Munich, Germany; judith.spiro@med.uni-muenchen.de; 5 Department of Radiology, University Hospital Cologne, D-50937 Cologne, Germany; daniel.pinto-dos-santos@uk-koeln.de

**Keywords:** artificial intelligence, medical imaging, machine learning, clinical translation, continuing medical education

## Abstract

Successful adoption of artificial intelligence (AI) in medical imaging requires medical professionals to understand underlying principles and techniques. However, educational offerings tailored to the need of medical professionals are scarce. To fill this gap, we created the course “AI for Doctors: Medical Imaging”. An analysis of participants’ opinions on AI and self-perceived skills rated on a five-point Likert scale was conducted before and after the course. The participants’ attitude towards AI in medical imaging was very optimistic before and after the course. However, deeper knowledge of AI and the process for validating and deploying it resulted in significantly less overoptimism with respect to perceivable patient benefits through AI (*p* = 0.020). Self-assessed skill ratings significantly improved after the course, and the appreciation of the course content was very positive. However, we observed a substantial drop-out rate, mostly attributed to the lack of time of medical professionals. There is a high demand for educational offerings regarding AI in medical imaging among medical professionals, and better education may lead to a more realistic appreciation of clinical adoption. However, time constraints imposed by a busy clinical schedule need to be taken into account for successful education of medical professionals.

## 1. Introduction

Artificial intelligence (AI) has become one of the dominant topics in medical research, especially in processing and analysis of medical imaging data [1,2]. This is documented by an ever-increasing number of research studies on AI in medical imaging, and various start-ups and established companies entering the medical-imaging market [3,4]. However, clinical adoption of AI algorithms for medical imaging is lagging behind for various reasons, such as a lack of clinical validation of AI algorithms, regulatory burdens, hesitance of patients to accept AI for individual clinical decisions, and as of yet, often unsatisfactory reimbursement for AI algorithms [3,4,5,6]. 

Another important reason may be that educational programs on AI in medical imaging tailored to the needs of medical professionals are lacking, which may lead to hesitance to use new algorithmic tools in clinical practice [7,8]. Very recently, some training programs for residents have been implemented into the formal radiology curriculum [9]. However, educational programs, which are open to a broader audience of medical professionals working with medical-imaging data, such as ophthalmologists or pathologists, are very rare.

To fill this gap, we created a 12-week, online-only course on AI in medical imaging and offered it for free to medical doctors (MDs) at our institution, and also to medical students and non-MD researchers. The overall goal of the course was to offer educational material on AI in medical imaging to healthcare professionals to give them a better appreciation of the underlying principles and so they could understand the potential pitfalls of using AI in clinical practice. The second point especially should lead to a better translation of imaging AI into clinical practice by reducing the commonly observed reservations of healthcare professionals to use AI in practice. Thus, the course material comprised the theoretical basics of AI in general, special challenges in medical imaging, basics of Python programming, and special-focus lessons highlighting particularly interesting fields of AI in medical imaging and its translation into clinical practice. 

In this article, we report on our initial experience with this educational program and how the participants perceived it. Furthermore, we assessed the participants’ opinions on AI in medical imaging, as well as their self-rated skills pertaining to the topic in order to inform other institutions seeking to develop educational programs for MDs in medical imaging.

## 2. Materials and Methods

### 2.1. Course Curriculum

The course was designed as a 12-week, online-only curriculum, consisting of two six-week blocks plus live online meetings before, during, and after the course. In the first block, the objective was to teach basics of AI in general and its applications in medical imaging. The study material was presented to the participants through an online teaching platform (Moodle) in a synchronous (i.e., live online lectures) and asynchronous manner (e.g., pre-recorded screencasts, reading assignments, and multiple-choice questions). Furthermore, there was an introduction to the concepts of Python programming, with dedicated examples based on Google Colab notebooks. Live lectures were held weekly at a fixed time, and were recorded for those who could not attend. The content was produced mostly by medical and non-medical researchers and lecturers from our institution in a standardized format. Topics of the first six weeks included “Introduction to Machine Learning: Historical Context, Systematic Considerations and Basics of Linear Algebra (part 1)” (week 1), “Introduction to Artificial Neural Networks: What Can AI Learn? and Basics of Linear Algebra (part 2)” (week 2), “Applying AI to Imaging: Special Considerations for Medical Imaging” (week 3), “Advanced Learning Methods with Artificial Neural Networks: Unsupervised Learning” (week 4), “Generative Adversarial Networks and Medical Image Formats” (week 5), and “Critical Appraisal of AI studies in Radiology: Reporting Metrics and Paper Analysis” (week 6). 

**Figure 1:**
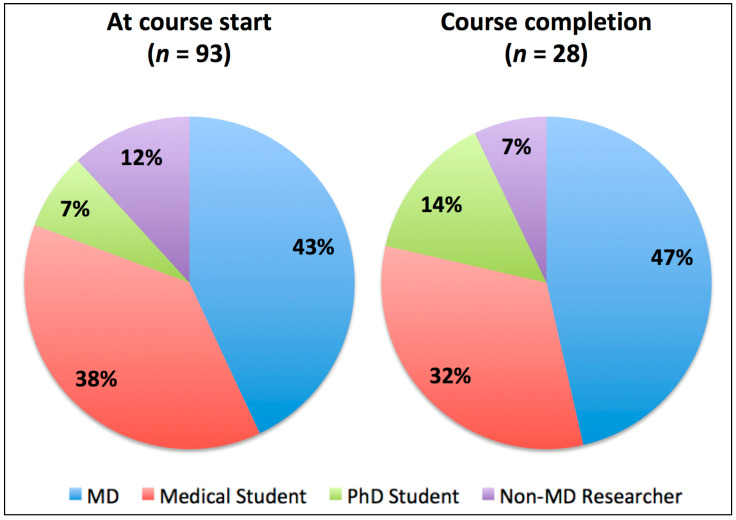
Pie chart diagrams showing the percentages of professional groups at course start (**left**) and after successful course completion (**right**).

The second block consisted of one special-focus lesson per week, highlighting a particular topic of applied AI. These lessons were prepared following a flipped-classroom concept with pre-recorded lectures, reading assignments, and a live question-and-answer session with the lecturer. The topics were the following: “Structured Reporting in Radiology” (week 7), “Explainable AI in Medical Imaging” (week 8), “Computational Pathology” (week 9), “AI in Dermatology” (week 10), “AI in Imaging Neuroscience: Ethical, Legal, and Societal Aspects” (week 11), and “Ethics in AI” (week 12). Accompanying the second half of the course, the participants were asked to perform a group work task, which consisted of the detailed analysis and presentation of a current research publication in the field of medical imaging AI. Numbers of participants per group before and after the course can be found in Table 1 and in Figure 1.

### 2.2. Pre- and Post-Course Questionnaires

Pre- and post-course questionnaires were individually administered to the participants through the online learning system. Answers were given either in a yes/no manner, based on a five-point Likert-scale evaluation or as free text. The input was saved anonymously. The questionnaires and survey results are depicted in Table 2, Table 3 and Table 4.

### 2.3. Statistical Analysis

Descriptive and comparative statistics were performed using SPSS version 26.0 (SPSS, IBM Corp. 2019, Armonk, NY, USA). Pre- and post-course evaluations were compared using Wilcoxon’s signed-rank test. Statistical significance was assumed for *p* < 0.05.

### 2.4. Ethics Statement

Participants consented to the statistical evaluation and potential publication of the evaluation results. Evaluation results were submitted anonymously. No confidential medical information was used in this study.

## 3. Results

### 3.1. Course Participants

In total, 93 participants (46 female (49.5%), 47 male (50.5%), mean age 29.6 ± 7.1 years) enrolled into the course and filled in the pre-course evaluation form. For post-graduates (*n* = 52), median time since graduation was 4.00 years (interquartile range: 2.00–8.75 years). The group of participants consisted of 40 medical doctors (MDs) (43.0%), 35 medical students (37.6%), 7 PhD students (7.5%), and 11 postgraduate researchers without an MD degree (11.8%). Within the group of medical doctors, eight specialized in neuroradiology, six in pathology, and three each in radiology, ophthalmology, nuclear medicine, neurology, internal medicine and dermatology. Two participating medical doctors specialized in psychiatry and surgery, and one each in pediatrics, neurosurgery, nephrology, and anesthesiology. Most participating medical doctors were residents (30; 75.0%), next to eight participating consultants (22.5%) and one head of department (2.5%). The majority of participants stated that they currently do not use AI in their daily work (77; 82.8%) and half of them had previous programming experience (46; 49.5%); 36 participants (39.2%) stated that they had no previous education in the field of artificial intelligence, while 40 (43.0%) had read some articles, and 16 (17.2%) had previously taken a course related to the topic. Overall, the participants planned to spend a median of 4.00 h per week on the course (IQR: 3.00–5.00).

A total of 47 (25 female (53.2%), 22 male (46.8%)) participants filled in the post-course evaluation, and 28 completed the course (16 female (57.1%) and 12 male (42.9%)), including 13 MDs, 9 medical students, 4 PhD students and 2 non-MD researchers (see Table 1).

### 3.2. Opinions towards AI in Medical Imaging

As part of the evaluation, we asked the course participants about their opinions and attitudes towards AI in medical imaging (see Table 2). Summarizing the results, the participants highly supported the statements that AI in medical imaging will benefit patients in the foreseeable future, that it will reduce the workload of physicians, and that it is already capable of performing particular, well-defined tasks at an expert-physician level. The majority of participants had the opinion that education about AI should be integrated into medical school and into residency, and that the doctor must understand how an algorithm works in order to use it on patients. Participants opposed the notion that AI will replace doctors, and were undecided on whether AI can perform image-analysis tasks in general at an expert-physician level. When it comes to obstacles for clinical adoption, there was a trend towards attributing this more to regulatory processes than to algorithmic performance per se.

**Figure 2:**
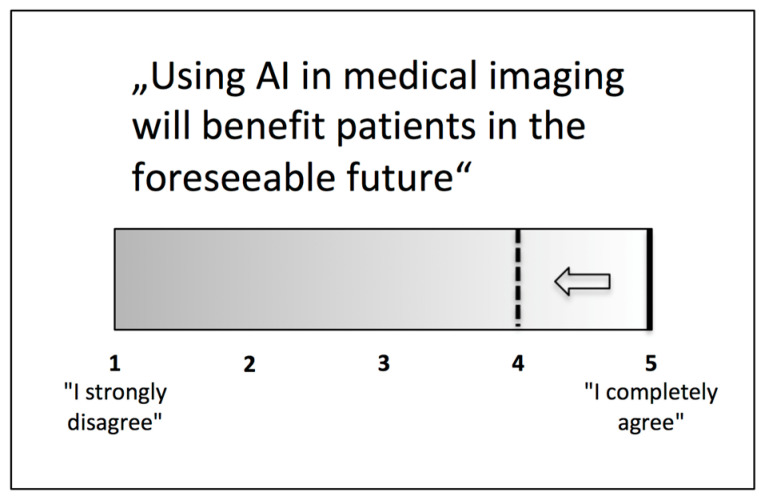
Median agreement with the statement “Using AI in medical imaging will benefit patients in the foreseeable future” changed from full to partial after the course.

In order to explore whether opinions on these topics changed after successful course completion, we analysed the participants’ answers before and after the course. There was a significant difference regarding their opinion on whether AI will lead to patient benefit in the foreseeable future, with less overoptimistic but still very positive answers after the course (pre-course evaluation: 5 (“I completely agree”); IQR (4.5–5.0); post-course evaluation: 4 (“I rather agree”); IQR (4.0–5.0); *p* = 0.020) (see Figure 2). With regard to the other questions, no significant change could be noted, other than a trend towards a slightly more affirmative opinion regarding the question “Some particular tasks can be performed by an AI algorithm today at medical-expert level” (*p* = 0.096).

### 3.3. Self-Perceived Skills Relating to AI and Medical Imaging

In order to assess whether the course impacted on skills regarding AI in medical imaging, we evaluated self-rated skills of those who successfully completed the course. For results in the particular areas of self-assessment, please see Table 3. In summary, self-perceived skills improved in all areas, for understanding Python code as well as for understanding concepts of linear algebra pertaining to AI. Furthermore, participants felt more confident to analyse a research paper in the field, to implement an AI algorithm in a clinical environment and to incorporate the decisions given by an algorithm into their clinical decision making.

### 3.4. Overall Appraisal of the Course

The participants were overall very satisfied with the study material and the organization of the course, and deemed the content of the course important for their work as a clinician or scientist. There was a small tendency to underestimate the time effort necessary for the course and towards the notion that taking the course alongside clinical work might be problematic. Most of the participants felt more competent at dealing with AI in medical imaging after the course. A majority of participants missed in-person events (which were not held due to the online-only character of the course). Please see Table 4 for details. 

## 4. Discussion

When evaluating this pilot educational program for medical professionals who wanted to study AI in medical imaging, we found that the interest in the medical community was very high. Furthermore, education about AI has the potential to change the opinions of medical professionals with regard to AI, and to improve their competencies pertaining to the topic. However, time constraints due to a busy schedule of clinical work impose a substantial hurdle for thorough education of medical doctors. 

The interest in our educational offering was very high among MDs and medical students from our institution, as expressed by the high number of enrolled participants at the beginning of the study. This was supported by results from recent surveys among radiologists: three-quarters of all participants in a survey (*n* = 270) from 2019 stated that they had received insufficient information about AI tools, and more than 90% would participate in continuing medical education offers on this topic [10]. The need for advanced teaching courses on AI in medical imaging was emphasized by results from a recent global survey among radiologists, in which only around 11% stated that they had advanced knowledge in the field [7]. In summary, our experience corroborated the high demand and need for educational offers for medical professions in the field of AI.

Our course participants had a rather positive opinion about AI in medical imaging, embracing its potential to perform defined tasks in image analysis, and thus to take workload off the current physician workforce. This could be explained by a positive selection bias, and fits with previous reports that openness for AI tools resulted in a more optimistic view on the topic and its impact on medicine [7]. 

Despite this optimism with respect to the impact of AI in medical imaging, most participants did not believe that AI would eventually replace physicians in a clinical environment. This was in line with a survey among medical students, who largely refuted the impression that radiologists would be replaced in the future [11]. The fear of replacement through new, AI-related technologies seemed more relevant in the largest currently available survey among radiologists, which found this to be of importance to almost 40% of participants [7]. Interestingly, this survey also proposed a potential explanation through an inverse correlation between AI-specific knowledge and fear of replacement through AI [7].

Although the attitude of MDs towards AI in medical imaging seems quite consolidated, we did find a significant change with respect to the question regarding whether AI will lead to patient benefits in the foreseeable future, with less-optimistic attitudes after the course. However, the answer to that question left room for interpretation, and did not differentiate whether the participants did not believe in patient benefits through AI at all, or just not in the foreseeable future. We interpreted this change in opinions as an indicator that our course and increased knowledge about AI among medical professionals impacts their attitude and judgment of clinical practicability, by highlighting not only the potential of AI (which is heavily discussed), but also discussing and teaching about the challenges and limitations of AI. 

Underlining the impact of the described course, we saw a significant increase in the self-assessed ratings of the participants’ skills pertaining to AI in medical imaging. Although this was an encouraging finding, and was also assessed in a similar way in previous studies [9], the subjective nature of these self-assessed ratings left room for some uncertainty. Future courses and educational programs should define a clear list of skills and capacities that participants will possess after the course, and (most importantly) a clear way to test those [12]. At best, these learning objectives will incorporate theoretical and practical skills based on broad consensus by medical schools, resident programs, and professional associations.

A clear downside of our course experience was the rather high drop-out rate. Based on the participants’ feedback, we did not attribute this to the quality of our course and the study material, but rather to the tight schedules of clinicians and medical students, which do not allow for easy integration of additional five to 10 working hours per week. However, as drop-out rates were comparable among the different participant groups, a busy schedule may not have been the only reason for participants to quit the course. Particularly for medical students, the fact that the course was not part of the regular curriculum and no mandatory credits had to be earned may have been of importance. For PhD students and non-MD researchers, a reason might be that the clinical orientation of course content did not fit their focus, as it was most likely unrelated to their work. We hypothesized that the drop-out rate could be reduced by integrating the course into a mandatory curriculum; e.g., in medical school or by setting a participation fee for doctors. These two measures could potentially increase the participants’ adherence to the schedule and their motivation to finish the course. Another aspect would be that the course curriculum could be adapted in order to avoid large leaps in complexity and to ensure step-wise learning without asking too much of the participants. Future studies should address how topics related to computer science and AI are best taught to medical professionals. Another point with a potential negative effect on the participants’ motivation was the online-only character of the course, which was mostly due to the current COVID-19 pandemic and restrictions on in-person meetings. We hypothesized that in-person meetings and the establishment of tighter personal relationships between students and teachers could also decrease the drop-out rate substantially.

## 5. Conclusions

In summary, we found that educational offerings on AI in medical imaging were regarded very well by medical professionals, leading to improved skills and (in part) to a less-optimistic perception of AI in medical imaging. In our opinion, this also showed that educating medical professionals on AI is feasible and may potentially contribute to a successful implementation of AI in clinical practice. However, time constraints of medical professionals may hinder successful course completion. Future efforts should aspire to clearly define learning objectives for medical professionals, and ideally to harmonize curricula integrated in medical schools and/or residency programs.

## Figures and Tables

**Table 1:** A list of the course participants according to group before and after the course.

Participant Group	MD	Medical Student	PhD Student	Non-MD Researcher
At course start	40	35	7	11
Successful course completion	13	9	4	2

**Table 2:** Questionnaire on the participants’ opinions on artificial intelligence in medical imaging and its clinical adoption. Results of the pre-course survey were given on a five-point Likert scale ranging from 1 = strong disagreement to 5 = complete agreement.

Question (Answers Ranging from 1 = Strongly Disagree to 5 = Completely Agree)	Median	Minimum	Maximum	25th Percentile	75th Percentile
Using AI in medical imaging will benefit patients in the foreseeable future.	5	3	5	4	5
It is important to understand how an AI algorithm works in order to use its results in clinical decision making.	5	2	5	4	5
I would use an AI algorithm in medical decision making if it has been thoroughly evaluated by others with good performance, although I don’t understand how it works.	4	1	5	3	4
I will not use AI in medical imaging algorithms unless I can fully explain them to my patients.	3	1	5	2	4
Education about AI must be integrated in medical training in university.	4	1	5	4	5
Education about AI must be integrated in medical training in residency.	4	1	5	4	5
Using AI in medical imaging will reduce the workload of physicians.	4	2	5	3	4
Clinical adoption of AI in medical imaging will replace physicians e.g., radiologists in the next 10 years.	2	1	5	1	3
Image-analysis tasks in general can be performed by an AI algorithm today at medical-expert level.	3	1	5	2	4
Some particular tasks can be performed by an AI algorithm today at medical-expert level.	4	2	5	4	5
Clinical adoption of AI algorithms in medical imaging is mostly hindered by regulatory barriers and traditions, not by the performance of the developed algorithms.	3	1	5	3	4
Doctors should have basic programming skills.	3	1	5	2	4

**Table 3:** Self-assessment of AI-related skills before and after the course shows significant improvement in all domains after the course.

Timepoint	Before Course			After Course			
Areas of self-Assessment (Ranging from 1 = No Skills to 5 = Expert Skills)	Median	25th Percentile	75th Percentile	Median	25th Percentile	75th Percentile	*p*
Understanding Python code when reading it.	1	1	2	2.5	2	3	0.001
Creating Python code for statistical analysis.	1	1	2	2	2	3	0.002
Understanding concepts in linear algebra pertaining to machine learning.	2	1.5	2	3	2	3.25	0.006
Assessing a machine-learning paper validating AI algorithms for medical imaging.	2	1	2	2.5	2	3.25	0.005
Applying a ML algorithm in a clinical setting.	1	1	2	2	2	2.25	0.013
Incorporating decisions made by a ML algorithm into clinical decision making.	1	1	3	2.5	2	3.25	0.042

**Table 4:** General course evaluation results given on a five-point Likert scale ranging from 1 = strong disagreement to 5 = complete agreement.

Question (Answers Ranging from 1 = Strongly Disagree to 5 = Completely Agree)	Median	Minimum	Maximum	25th Percentile	75th Percentile
The course was well organized	5	2	5	4	5
Overall, the study material was well prepared	5	3	5	4	5
The content of the course was important for my work as a clinician	3,5	2	5	3	4
The content of the course was important for my work as a scientist	4	2	5	4	5
The course could easily be taken alongside clinical work	3	1	5	2	3
I expected the workload to participate in the course to be	3	2	3	2	3
I missed in-person lectures and meetings with teachers and other students.	4	1	5	2	4
I feel more competent at dealing with AI in medical imaging than before the course	4	1	5	4	5

## Data Availability

Original data are stored by the authors and available upon reasonable request.
